# The Significance of FDG PET/CT–Derived Parameters in Determining Prognosis of Cases with Pancreatic Adenocarcinoma: A Prospective Study

**DOI:** 10.3390/medicina58081027

**Published:** 2022-08-01

**Authors:** Hwaida M. Mokhtar, Amira Youssef, Tamer M. Naguib, Amr A. Magdy, Samir A. Salama, Ahmed M. Kabel, Nesreen M. Sabry

**Affiliations:** 1 Radiodiagnosis Department, Faculty of Medicine, Tanta University, Tanta 31527, Egypt; hwaidamahmoudhm18@gmail.com; 2 Clinical Pathology Department, Faculty of Medicine, Tanta University, Tanta 31527, Egypt; damirayoussef@yahoo.com; 3 Anesthesia and ICU Department, Faculty of Medicine, Tanta University, Tanta 31527, Egypt; tnaguib1eg@yahoo.com (T.M.N.); amrmagdy@med.tanta.edu.eg (A.A.M.); 4 Division of Biochemistry, Department of Pharmacology, College of Pharmacy, Taif University, P.O. Box 11099, Taif 21944, Saudi Arabia; s.salama@tu.edu.sa; 5 Department of Pharmacology, Faculty of Medicine, Tanta University, Tanta 31527, Egypt; 6 Clinical Oncology Department, Faculty of Medicine, Tanta University, Tanta 31527, Egypt; nesreensabry1eg@yahoo.com

**Keywords:** pancreatic adenocarcinoma, PET/CT scan, matrix metalloproteinases, prognosis

## Abstract

*Background and objectives*: Pancreatic adenocarcinoma represents one of the common malignancies with a relatively poor prognosis. However, early detection of this type of cancer may prove to be curable. Recent advancements in the radiological techniques might represent a hope for the early diagnosis and prediction of prognosis of pancreatic adenocarcinoma. This study aimed to assess the prognostic value of the primary tumor volumetric parameters obtained from FDG PET/CT first stage for the overall survival (OS) and progression-free survival (PFS) of patients with pancreatic adenocarcinoma and to explore the possible correlation between serum matrix metalloproteinase-2 (MMP-2) and the patients’ characteristics. *Methods*: Fifty patients with pancreatic adenocarcinoma were subjected to FDG PET/CT scan. The SUVpeak, SUVmax, and the metabolic tumor volume (MTV) were determined, as well as the SUVmean of the liver. Moreover, serum levels of MMP-2 were assessed. Follow-up of the patients was carried out for sixty months with determination of PFS and OS. *Results*: Peak SUV ≥ 3.9 was significantly correlated with the primary pancreatic lesions’ mean total glycolytic activity of >92 g, and MTV and was directly correlated with mortality. There was a positive correlation between peak SUV ≥ 3.9 and 50% SUVmax threshold > 82. Moreover, there was significant correlation between the total glycolytic activity and the studied clinicopathologic factors, except the age and sex of the patients and ECOG performance status. In addition, FDG uptake and the tumor glycolytic activity were substantially linked with a shorter PFS. Similarly, a strong correlation was found between MTV and PFS. Serum MMP-2 levels showed a significant relationship with the performance status, tumor stage, SUVmax threshold, and the glycolytic activity. *Conclusions*: Peak SUV, main lesion SUVmax, serum MMP-2, and the tumor glycolytic activity are good predictors of PFS of patients with pancreatic adenocarcinoma.

## 1. Introduction

Pancreatic cancer is regarded to be the seventh leading cause of cancer-related mortality globally, as it is rapidly progressive with poor prognosis even if discovered early [1]. Because patients seldom exhibit symptoms until an advanced stage of the disease, the mortality rate is rapidly increasing worldwide [2]. Despite advancement in diagnosis and management of pancreatic cancer, the five-year survival rate remains at about 9% [3].

The mortality rate incidence of pancreatic malignancies is increasing rapidly in developing countries owing to massive smoking, dietary factors, pancreatitis, physical inactivity, and genetic mutations [4]. Malignant pancreatic tumors include pancreatic exocrine tumors mainly adenocarcinoma, neuroendocrine tumors, mucinous tumors and other rare forms of lymphoma [5].

Ultrasonography has a limited role in the imaging of pancreatic carcinoma due to the presence of bowel gases which often hinder the visualization of the pancreas. In contrast], multi-detector computed tomography (MDCT) and magnetic resonance imaging were the best for patients with suspected pancreatic cancer [6]. They help in the diagnosis and evaluation of local tumor invasion, invasion of the vascular structures, and distant spread [7].

Recently, the positron emission tomography/computed tomography (PET/CT) scan emerged as an important tool for pancreatic cancer management. Several studies have shown that functional imaging with F-18 fluorodeoxyglucose-positron emission tomography (^18^F-FDG PET/CT) has a greater specificity and sensitivity than the traditional imaging for pancreatic adenocarcinoma staging [8,9]. For instance, FDG PET/CT shows higher sensitivity and specificity to specific cancers and has been involved initially in staging and restaging to guide the physicians for the appropriate patient’s care. Additionally, it can distinguish between patients who will respond well and those who will not respond prior to occurrence of any tumor reduction [1,2,3].

Recent studies have pointed to the ability of FDG PET/CT imaging for a better delineation of radiotherapy planned dose, compared with CT scan in the treatment of advanced pancreatic adenocarcinoma [10]. Furthermore, several studies have established a median difference in tumor maximum standardized uptake values (SUVmax) based on pre- and post-treatment FDG PET/CT to accurately predict progression-free survival (PFS) and the overall survival (OS) in patients treated with definitive chemoradiation therapy for pancreatic adenocarcinoma [11].

Strong desmoplastic reactions and early metastasis are considered as the cornerstones for tumor invasion in pancreatic cancer. Proteolytic degradation of extracellular matrix (ECM) components can trigger tumor growth. ECM is proven to be a crucial source for cell binding proteins and growth factors affecting tumor cell behavior. [12] Matrix metalloproteinases (MMPs) have many subtypes including MMP2 which belongs to the gelatinase family. MMP2 is involved in the degradation of collagense IV/V in ECM via their proteolytic functions. The overexpression of MMP2 in cancer causes the decomposition of ECM and, hence, accelerated cancer invasion and worse prognosis [13]. MMP-2 expression was reported to be upregulated in pancreatic cancer and was proven to have positive correlation with the stage and grade of the tumor and with the chances of tumor recurrence [14].

The objective of this study was to assess the prognostic value of the primary tumor volumetric parameters obtained from FDG PET/CT first stage for the overall survival (OS) and progression-free survival (PFS) of pancreatic adenocarcinoma patients. Another objective of this work was to assess the correlation between serum levels of MMP-2 and the patients’ characteristics.

## 2. Patients and Methods

### 2.1. Patients

Between June 2015 and May 2020, fifty patients referred to Clinical Oncology department, Tanta University Hospital, Tanta, Egypt with pathologically confirmed adenocarcinoma of the pancreas were included in this study. All patients who had initial FDG PET/CT staging at Radiology Department, Tanta University Hospital were included starting from June 2015 to the end of the study in May 2020.

#### 2.1.1. Inclusion Criteria

- Patients aged between 18 and 70 years.
- Patients who had radiotherapy and chemotherapy.
- Patients who had initial FDG PET/CT staging images with an Eastern Cooperative Oncology Group (ECOG) performance status of 0–2.
- Patients who had adequate bone marrow reserve (WBCs count 3.5 × 10^9^/L, ANC count 1.5 × 10^9^/L, platelets 100 × 10^9^/L, and hemoglobin 10 g/dL).
- Patients who had normal renal functions (measured creatinine clearance 60 mL/min).
- All patients had histologic or cytologic evidence of pancreatic adenocarcinoma.

#### 2.1.2. Exclusion Criteria

- Patients with severe arrhythmia, prior surgery, peripheral neuropathy, chemotherapy or radiotherapy, symptomatic heart failure, active infection.
- Pregnant or lactating mothers.
- Patients with other malignant diseases.
- Patients with other comorbid diseases.

Medical records of all patients were properly revised, organized, and analyzed.

### 2.2. Intervention

This prospective, single-arm study was conducted at a single institution. The Ethical Committee of Faculty of Medicine, Tanta University, Egypt approved this study.

#### 2.2.1. Pre- and on-Treatment Evaluation

Monitoring of pre- and on-treatment consisted of physical examination, routine laboratory studies, medical history, initial FDG PET/CT staging images, cancer antigen 19.9 (CA19.9), and carcinoembryonic antigen (CEA) measurement.

#### 2.2.2. PET/CT Protocol

All patients fasted for at least 6 h prior to scanning. Patients were instructed to eat a high protein and low carbohydrate diet 24 h before the examination. Their blood glucose levels, height, and weight were recorded at the PET study time. Patients had a mean blood glucose level of 113.9 mg/dL (ranged from 60–193 mg/dL), and renal function tests were recorded. An intravenous cannula was inserted for 18F-FDG administration.

Patients were asked to avoid any stressful conditions to avoid physiological tracer uptake by the active muscles. One and half litres of water, acting as a neutral oral contrast agent, were consumed by the patients one hour before the examination. After that, patients were manually injected with FDG using (1.3 × 0.2 mCi/kg [48.1 × 7.4 MBq/kg] a weight-based formula, with a mean dosage of 15.7 mCi (580.9 MBq). Following an incubation period of 64.1 min on the average, low dose enhanced CT scan was done following injection of 1–2 mL/kg of ultravist at a rate of 4 mL/s by using an automatic injector, with the following parameters: 130 kV, 100 mA, 1-s tube rotation, 4-mm slice thickness, and bed speed of 8 mm/s. mA, 1-s tube rotation, 4-mm slice thickness, and bed speed of 8 mm/s. One case developed a minor reaction to the contrast and was controlled by I.V corticosteroids.

All of the patients’ bodies were scanned from the base of their skulls to their midthighs. The following parameters were used: 140 kV, 60 mA, 5 mm slice thickness, and 0.5 mm incrimination. The duration of the scan was approximately 25–35 min. PET scan immediately followed the CT study without moving the patient. The reconstructed helical PET and CT images were then re-formatted into axial, coronal and sagittal images of the body. Fusion images of PET and CT data were obtained by integrating the two data types. For attenuation correction, the PET image data sets were reconstructed using CT data, and the co-registered scan images were shown and assessed on the workstation using a special software.

#### 2.2.3. Image Analysis

The SUVpeak, SUVmax, and MTV of the tumors were determined, as well as the SUVmean of the liver [15]. The SUVmax was calculated using a spherical volume of interest (VOI) analysis in the sagittal, axial, and coronal planes that encompassed the entire tumor. It was ascertained that the VOI does not include organs that typically absorb 18F-FDG, such as the colon and stomach. As a baseline, the following PET-related metrics were used: The SUVmax of a tumor, which is defined as the SUV at the maximum tracer uptake (the hottest voxel); the SUVpeak of a tumor, which is defined as the average SUV within a 1-cm^3^ spherical volume surrounding the SUVmax; the SUVmean of the liver (as a reference organ); the MTV, which is defined as the volume of the tumor that exhibited FDG uptake; and the TBR, which is defined as the ratio of the tumor’s SUVmax to the SUVmean of the healthy liver tissue. To accomplish this, images of the tumor were segmented using a fixed-threshold technique, and an SUVmax threshold value of >2.5 was used to detect the tumor, as previously demonstrated for its simplicity and objectivity in multiple previous studies. The total lesion glycolysis (TLG) was calculated by multiplying the SUVmean of the tumor by the MTV [15,16].

#### 2.2.4. Measurement of Serum MMP-2 by ELISA Technique

Four milliliters of blood were collected on a dry vacutainer for performance of the laboratory assays then left to clot and centrifuged at 3000 revolution per minute (rpm) for 10–20 min. Aliquots of serum were prepared and immediately frozen at −20 °C for use to determine serum MMP-2 levels. Enzyme-linked immunosorbent assay (ELISA) detection was carried out using Quantikine TM total MMP-2 immunoassay kit (Catalog Number MMP200, R&D Systems, Minneapolis, MN, USA) which utilizes a pre-coated microplate with a monoclonal antibody specific for total MMP-2. The wells were pipetted with standards and samples, and the immobilized antibody binded to any MMP-2 present. After eliminating any binding chemicals, the wells were treated with a polyclonal antibody directed against total MMP-2. After eliminating any unbound antibody-enzyme reagent, the wells were filled with a substrate solution, and the color developed in proportion to the amount of the total MMP-2 bound in the initial phase. Then, the color’s development was stopped, and its intensity was determined.

#### 2.2.5. Treatment Protocol

##### Surgery

Three patients (6% of the study population) were evaluated for surgery based on review of FDG PET/CT imaging, which indicated that the disease was technically resectable. They had pancreaticoduodenectomy performed.

##### Chemotherapy

Chemotherapy was administered biweekly to 15 patients in the form of FOLFIRINOX. For patients without progressive disease (PD) or severe toxicity, therapy was maintained for as many as 12 months of treatment over the course of six months. All patients received appropriate hydration, anti-emetic medications, and corticosteroids. Patients were treated with growth factors and antibiotics based on expert clinical judgement in these cases.

##### Dose Adjustment of FOLFIRINOX

Every two weeks, decisions were made on whether to alter chemotherapy doses, discontinue treatment, or continue with the course. If the absolute granulocyte count (AGC) was greater than 1000 cells/L, platelets were greater than 100,000 cells/L, and non-hematologic toxicities were grade 2, the full biweekly doses of FOLFIRINOX were delivered. If AGC was between 500 and 1000 cells/L or the platelet count was between 50,000 and 100,000 cells/L, the dosage of FOLFIRINOX was reduced by 25%. The dosage of FOLFIRINOX was lowered by 50% to avoid grade 3 non-hematologic adverse effects. If AGC count was 500 cells/L, the platelet count was 50,000 cells/L, and/or the grade 4 non-hematologic adverse effects occurred, toxic consequences were present.

##### Chemoradiotherapy

A total of 32 (64%) patients received neoadjuvant chemoradiotherapy. All patients had residual tumors on subsequent evaluation, with 3 patients having reduction in the tumor size to the extent of becoming eligible to pancreaticoduodenectomy after radiation therapy. The remaining 29 patients (58%) had continued chemoradiation therapy.

A protocol-based concurrent gemcitabine–IMRT external beam radiation therapy was delivered to 32 patients (64%) at 2.0 Gy per fraction, to a total dose of mean planning target volume of 50.0 Gy if possible, in 25 fractions. Gemcitabine was administered on days 1, 8, 22, and 29 (1000 mg/m^2^ infused over 100 min).

The radiotherapy field for IMRT encompassed the gross tumor volume (GTV) included primary tumor and regional involved lymphatics identified on the pretreatment FDG PET/CT scan, including the celiac axis, superior mesenteric vessels, and porta hepatis. The CTV included the GTV plus a 0.5 cm. The planning target volume (PTV) included the CTV plus 0.5 cm.

#### 2.2.6. Evaluation during Concurrent Gemcitabine—IMRT External Beam Radiation Therapy

During the course of therapy, patients had a guided history and physical examination every week to monitor their progress. Any adverse events that occurred were documented, as well as their nature. Before each dosage of gemcitabine, complete blood count was performed. Blood levels of bilirubin, aspartate transaminase, alkaline phosphatase, electrolytes, blood urea nitrogen, alanine transaminase, phosphorus, albumin, total protein, creatinine, calcium, and glucose were clinically assessed. One month after completion of protocol-based concurrent gemcitabine–IMRT external beam radiation therapy, treatment monitoring consisted of CT-scan of the abdomen and pelvis. Patients who didn’t develop PD or had unacceptable toxicities were maintained on therapy for additional two cycles of gemcitabine infusions to complete neoadjuvant treatment.

#### 2.2.7. Restaging

After completion of treatment, patients were evaluated by FDG PET/CT of the body to radiographically document tumor response. Three individuals were considered for surgery when their disease was technically resectable upon treatment completion.

### 2.3. Outcomes

The endpoints of the present study were PFS and OS and their correlation with FDG PET/CT–derived parameters and serum MMP-2. From the commencement of treatment, PFS was calculated. Serial axial CT imaging was used to define objective local progression as any indication of an increase in the size of the original pancreatic tumor. Serial axial CT imaging was used to define distant progression as the unmistakable emergence of distant metastatic illness.

### 2.4. Time Frame

This study started from June 2015 to May 2020. The overall-survival (OS) rates were calculated from the time of the initial treatment to the time of the last follow-up visit or death using the Kaplan–Meier method [16].

### 2.5. Statistical Analysis

The obtained results were analyzed by the Statistical Package for the Social Sciences (SPSS) version 26 (IBM Inc., Chicago, IL, USA). Quantitative variables were presented as mean ± standard deviation (SD). Comparison between the two groups was carried out utilizing unpaired Student’s *t*-test. Qualitative variables were presented as frequency and percentage (%) and were analyzed utilizing the Chi-square test or Fisher’s exact test when appropriate. Kaplan-Meier curve was used to show the survival. Cox-regression analysis was used to estimate odds of recurrence and its 95% confidence interval on univariate and multivariate levels and to evaluate the independent prognostic variables affecting OS and PFS. A two tailed *p*-value < 0.05 was considered statistically significant.

## 3. Results

### 3.1. Patients’ and Tumors’ Characteristics

The present study was conducted on 50 patients with adenocarcinoma of the pancreas, with their age ranging from 30 to 72 years at the time of diagnosis (mean 53.2 years; SD ± 16.1). Their tumors’ sizes ranged between 1.5 and 30 cm. The majority of cases were stage III or greater, node positive, and grade III. Follow-up continued for sixty months. Patients’ characteristics as well as their relation to FDG uptake in the primary pancreatic tumors are summarized in Table 1.

### 3.2. FDG PET/CT Parameters Results

Table 1 summarizes the relation of FDG uptake in the primary pancreatic tumor (peak SUV < 3.9 vs. ≥3.9) to the patient and tumor characteristics, as well as to treatment data, and mortality. Twenty-four cases (48%) showed the primary pancreatic lesion’s mean total glycolytic activity of >92 g and 26 cases (52%) showed the primary pancreatic lesion’s mean total glycolytic activity of ≤92 g. The metabolic tumor volume was <8 cm^3^ in most of the cases (Table 1). There was significant correlation with grade, with a higher frequency of grade III cancers having peak SUV, ≥3.9 (*p* < 0.001). Peak SUV, ≥3.9 was significantly correlated with the primary pancreatic lesion’s mean total glycolytic activity of >92 g, and metabolic tumor volume (all *p* = 0.001) and directly correlated with mortality. There were also positive correlations between peak SUV, ≥3.9 and 50% SUVmax threshold >82. There was no statistically significant correlation when observing the effect of peak SUV, ≥3.9 on the ECOG performance status and age or sex of the patients.

### 3.3. Tumor Glycolytic Activity

By using gradient edge detection, a cutoff point of 92 g for median tumor glycolytic activity was found to be higher than 92 this value in 24 patients (48%). Table 2 summarizes the relation of tumor glycolytic activity to the patient and tumor characteristics as well as to treatment modality and mortality. The table shows statistically significant correlation with all the studied clinicopathologic factors except the age and sex of the patients and ECOG performance status.

**Figure 1:**
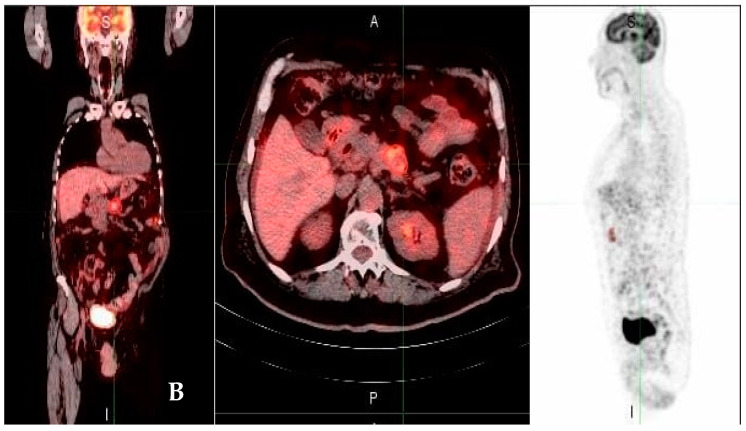
A 66-year-old man was diagnosed with stage II pancreatic adenocarcinoma (T3N0M0). PET/CT scans performed at baseline reveal minor metabolic activity (yellow regions, (**A**,**B**); maximum standardized uptake value, 3.7) consistent with primary pancreatic mass; projection of maximal intensity PET imaging demonstrates 33 mL of tumor glycolytic activity without signs of metastatic involvement (pancreatic body mass).

**Figure 2:**
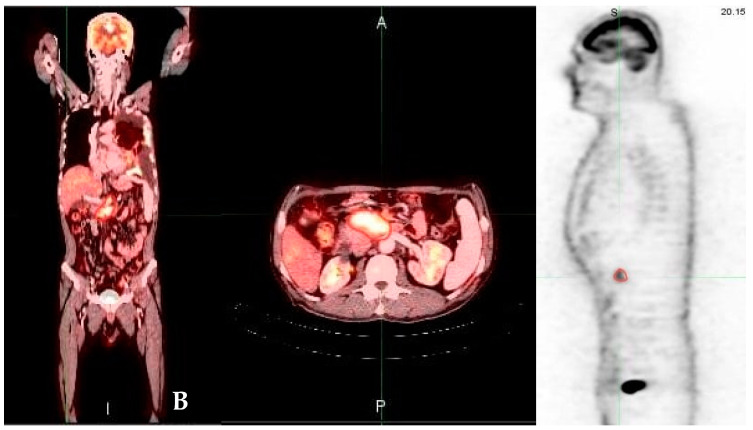
A 57-year-old man with pancreatic adenocarcinoma stage four. PET/CT scans performed at baseline reveal moderate metabolic activity (yellow regions, (**A**,**B**); maximum standardized uptake value, 6.6) consistent with primary pancreatic mass; projection of maximal intensity PET scan demonstrates 227 mL of tumor glycolytic activity with signs of metastatic involvement (pancreatic head mass with superior mesenteric lymph nodes and left parietal pleura of high metabolic activity).

SUV showed positivity in all cases (100%). Peak SUV was <3.9 in 24 cases (Figure 1), and ≥3.9 in 26 cases (Figure 2).

### 3.4. Relationship to Survival

**Figure 3:**
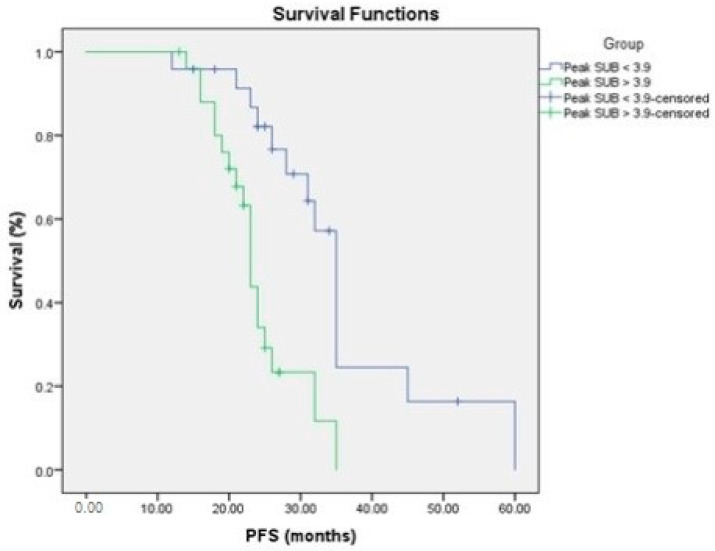
PFS in relation to FDG uptake.

**Figure 4:**
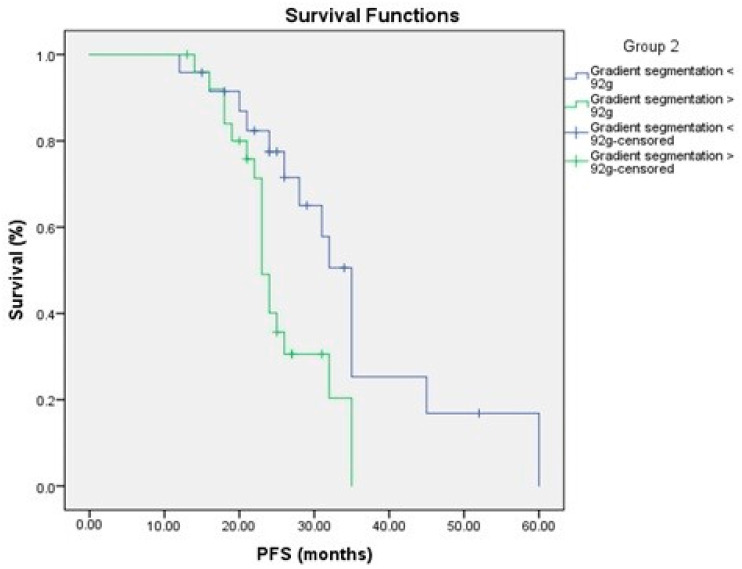
PFS in relation to the glycolytic activity.

To determine the prognostic importance of FDG uptake in primary pancreatic tumors and tumor glycolytic activity, we compared FDG uptake in primary pancreatic tumors and tumor glycolytic activity to PFS and OS. FDG uptake in the main pancreatic tumor and tumor glycolytic activity were substantially linked with a shorter PFS in a univariate analysis (Figure 3 and Figure 4, Table 3 and Table 4).

Similarly, univariate analysis revealed a strong correlation between metabolic tumor volume and PFS. On the other hand, in univariate analysis, sex, age, stage at diagnosis, and type of therapy were not significantly associated with PFS. Only primary pancreatic tumor glycolytic activity was shown to be independently associated with this end point in multivariate analysis.

**Figure 5:**
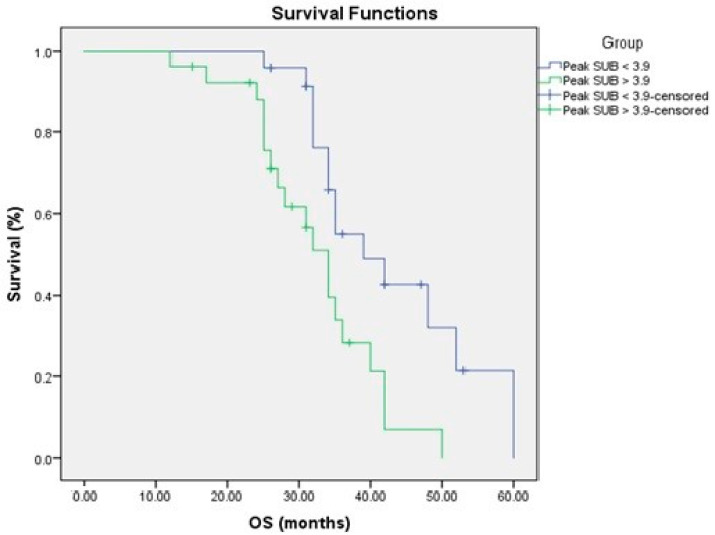
The overall survival in relation to FDG uptake.

**Figure 6:**
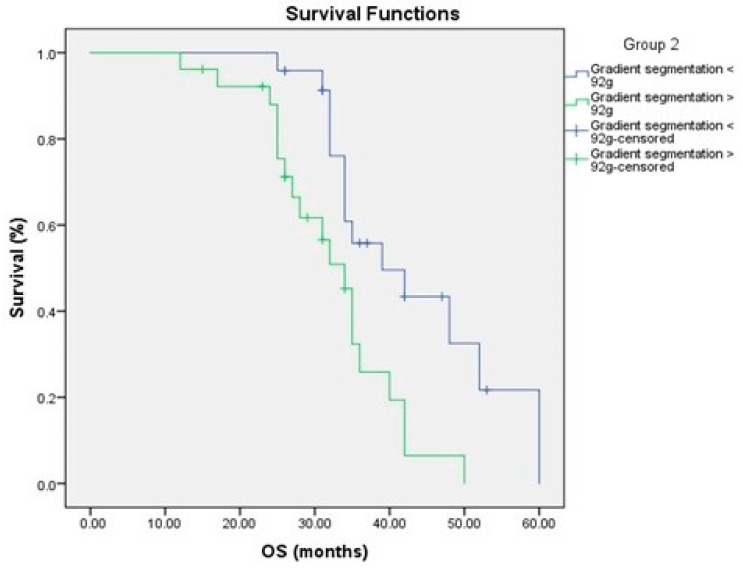
The overall survival in relation to the glycolytic activity.

In terms of OS, the most important prognostic factors were tumor glycolytic activity, peak SUV and SUV max (Figure 5 and Figure 6, Table 5 and Table 6).

Kaplan–Meier survival curves showed better prognosis of low tumor glycolytic activity <92 g, SUV max < 6.5 and peak SUV < 3.9 for PFS and OS (Figure 4 and Figure 6).

### 3.5. Serum MMP-2 Levels

MMP-2 serum marker showed significant relationships with performance status, tumor stage, the SUV max threshold, and the glycolytic activity but did not show any significance with age or grade (Table 7).

## 4. Discussion

Pancreatic cancer is world’s seventh largest cause of cancer death, with a five-year survival rate of about 7% [1]. The correct diagnosis of pancreatic cancer is critical for selecting the most appropriate treatment strategy. ^18^F-FDG PET/CT had emerged as a powerful imaging technology for finding and monitoring various malignancies, and it is used to stage, detect local recurrence as well as distant metastases, monitor therapeutic effects, and predict prognosis in various malignancies, including pancreatic cancer [17,18].

Recent studies suggested that measures generated from FDG PET/CT, such as SUV, MTV, and TLG, can be used to predict a variety of cancers, including pancreatic malignancies. However, there is no agreement on the optimum criteria for assessing patients’ prognosis, operability, and the other prognostic factors [19,20,21].

According to our study results, peak SUV and SUV max were associated with worse prognosis of pancreatic cancer (*p* < 0.05). Additionally, high levels of total glycolytic activity and metabolic tumor volume showed significant association with mortality (*p* < 0.05). This result is in the line with the work of Choi et al. [22], where locally advanced pancreatic cancer in those who have been treated with chemotherapy showed significantly low SUVmax, MTV, or TLG (*p* < 0.05) and was strongly correlated with longer OS. Moreover, MTV and TLG showed significant prediction ability for PFS, locally advanced pancreatic cancer, as well as OS (*p* < 0.05) [23,24,25,26,27,28,29,30]. Another study by Wu et al. [26] revealed that that MTV was also an independent prognostic factor for those with pancreatic cancer. According to Lee et al. [31], 87 pancreatic carcinoma patients with surgical resection showed a correlation between TLG and MTV which helped in the prediction of OS and RFS (*p* < 0.05). In the same line, Xu et al. [32] found that MTV and TLG were independent risk factors in 122 patients with resectable pancreatic ductal carcinoma. In the contrary to the previous studies’ conclusion, Hyun et al. [21] found that TLG was not an independent prognostic factor. Nevertheless, Moon et al. [25], Davison et al. [28], and Chang et al. [29] exploited the hybrid imaging biomarker for a variety of different cancers. They determined that this hybrid was a more accurate predictor of patient outcome than tumor SUVmax or computed tomography tumor measures.

In the present study, age did not show significant association with SUV or the performance status (*p* > 0.05). This matches with the findings of Hyun et al. [21], where 137 cases with pancreatic ductal adenocarcinoma showed insignificant association with the age of these patients (*p* > 0.05). However, FDG PET/CT was able to predict recurrence of pancreatic ductal adenocarcinoma within one-year. Moreover, the study by Choi et al. [22] showed no significant correlation between SUV activity and patients’ characteristics, such as age, sex, and performance status.

The majority of cases in the current study were node positive at stage III or higher. Peak SUV was 3.9 in 24 instances and 3.9 in 26 cases with a significant association between grade and peak SUV (*p* < 0.05). Grade III tumor exhibited a higher frequency of 3.9 (*p* < 0.001). Similarly, Casneuf et al. [23] and Heinrich et al. [24] studied the impact of using 18 FDG PET/CT in staging and therapy planning of pancreatic cancer. Both studies revealed a significant correlation between SUV and tumor stage, grade, and lymph node status. In the same line, Moon et al. [25] included 21 patients with locally advanced pancreatic cancer and found a correlation of the SUV value and tumor size. This study has justified these results as it may be due to tumor hypercellularity.

Furthermore, Sun et al. [27] had 91 patients with pancreatic cancer all diagnosed using FDG PET/CT before therapy. Using the best cut-off value of SUV = 5.49, they divided all patients into two groups: High and low SUV groups (SUV > 5.49) and (SUV < 5.49). The study showed that SUV in patients with cancer pancreas was only associated with tumor size (*p* < 0.05). Additionally, the other clinical and pathological features of all included patients did not correlate with SUV values whether high or low, which matches the findings of the current study.

In this study, gradient edge detection was used to assess tumor glycolytic activity, and the cut-off point of 92 g was determined to be >92 g in 24 cases (48 percent). It showed statistical significance with the measured clinicopathologic parameters except for age and sex as well as patients’ ECOG performance status (*p* < 0.05).

The glucose metabolic phenotype was reported to play a role in the initiation and the progression of pancreatic cancer [33]. The cells of pancreatic adenocarcinoma exhibited suppression of aerobic glycolysis with less energy yield which was associated with enhanced tumor progression and poor prognosis [34]. Therefore, the novel onco-immunology therapies of pancreatic cancer are directed towards the glycolytic pathways [35]. In the present study, the FDG uptake in the tumor glycolytic activity was analyzed in relation to PFS and OS. FDG uptake was significantly associated with shortened PFS in the primary pancreatic tumor and tumor glycolytic activity (*p* < 0.05). Similarly, the metabolic tumor volume was only significantly associated with PFS (*p* < 0.05). This agrees with the results of Ren et al. [7]., Arnone et al. [8], and Topkan et al. [9], where FDG PET/CT was associated with the detection of PFS. In terms of OS, the most important prognostic factors were tumor glycolytic activity, peak SUV, and SUV max. Kaplan–Meier survival curves demonstrated better prognosis of low tumor glycolytic activity < 92 g, SUV max < 6.5 and peak SUV < 3.9. Similarly, Schellenberg et al. [30] studied 55 unresectable pancreatic cancer patients who received stereotactic body radiation therapy, where SUVs and molecular tumor boards from PET scans were independent predictive variables for both OS and PFS. Similarly, Choi et al. [36] reported that a SUVmax of 3.5 of a primary tumor was an excellent predictive factor for OS and PFS in 64 patients who received curative pancreatic carcinoma resection. In the investigations of Nakata [37], tumor FDG uptake exceeding the SUVmax of 3.0 was associated with poor prognosis, although only in patients with incurable sickness, not in those who received surgical resection. Similarly, Sperti et al. [38] studied 60 patients with atypical pancreatic adenocarcinoma to reveal that primary tumor FDG uptake below median cut-off SUVmax of 4 was associated with a significantly longer OS (265 days) than tumor FDG uptake beyond this median cut-off SUVmax of 4 (178 days).

The results of the present study revealed that a higher serum level of MMP-2 was associated significantly with tumor staging (*p* = 0.043), 50% SUVmax threshold (*p* = 0.003), and ECOG performance status (*p* = 0.019). This was in consistent with many previous studies where an association between MMP2, pancreatic adenocarcinoma invasion, and metastasis was detected [39]. Interestingly, MMPs were reported to be associated with genetic alterations, especially the K-Ras mutation, which plays a crucial role in the progression of pancreatic dysplastic lesions into pancreatic adenocarcinoma [40]. Moreover, the enhancement of Kras-induced MMPs expression by gemcitabine was proven to be responsible for gemcitabine-resistant pancreatic tumor cells [41]. Javadrashid et al. [42] indicated that the tumor microenvironment is one of the challenges that prevent the chemotherapeutic agents from attacking the pancreatic tumor cells, enabling these cells to invade the immune system. MMPs were proven to shape the tumor microenvironment to be more suitable for tumor invasion and propagation [43].

## 5. Conclusions

Peak SUV, main lesion SUVmax, serum MMP-2, and the tumor glycolytic activity can be considered as good predictors in pancreatic adenocarcinoma patients’ PFS. Regardless of the clinical variables, the tumor glycolytic activity, when evaluated by baseline PET, showed that it was a strong predictive imaging parameter for OS.

### Limitations of the Study

This study had a limited access to larger number of patients, which rendered a confirmed conclusion regarding possible other treatment options in the case of a worse prognosis observed with the mentioned prognostic variables in FDG PET/CT. Moreover, a future comparison of the effects of different treatments and the impacts of FDG PET/CT on the enhancement of prognosis of the different types of pancreatic malignancies is required.

## Figures and Tables

**Table 1:** FDG uptake in the primary pancreatic tumor in relation to patient and tumor characteristics as well as to treatment modality, glycolytic activity and mortality.

		FDG Uptake in the Primary Pancreatic Tumor						Chi-Square	
		Peak SUV < 3.9		Peak SUV ≥ 3.9		Total			
		N	%	N	%	N	%	*X* ^2^	*p*-Value
Age	<60	10	20%	11	22%	21	42%	0.012	0.963
	>60	14	28%	15	30%	29	58%		
Sex	Male	11	22%	17	34%	28	56%	1.938	0.164
	Female	13	26%	9	18%	22	44%		
ECOG performance status	0	3	6%	1	2%	4	8%	1.271	0.260
	1	21	42%	25	50%	46	92%		
Tumor Stage	II	3	6%	0	0%	3	6%	3.928	0.140
	III	11	22%	11	22%	22	44%		
	IV	10	20%	15	30%	25	50%		
Tumor Grade	Grade I	0	0%	0	0%	0	0%	6.462	0.011 *
	Grade II	14	28%	6	12%	20	40%		
	Grade III	10	20%	20	40%	30	60%		
Therapy	None	0	0%	3	6%	3	6%	6.029	0.110
	chemotherapy	7	14%	8	16%	15	30%		
	chemoradiotherapy	14	28%	15	30%	29	58%		
	Chemoradiotherapy + surgery	3	6%	0	0%	3	6%		
glycolytic activity	gradient segmentation > 92 g	4	8%	20	40%	24	48%	18.147	0.001 *
	gradient segmentation ≤ 92 g	20	40%	6	12%	26	52%		
50% SUVmax threshold	>82	2	4%	21	42%	23	46%	26.631	0.001 *
	≤82	22	44%	5	10%	27	54%		
metabolic tumor volume	<3 cm^3^	9	18%	1	2%	10	20%	18.486	0.001 *
	≥3 cm^3^–5 cm^3^	10	20%	6	12%	16	32%		
	>5 cm^3^–8 cm^3^	5	10%	9	18%	14	28%		
	>8 cm^3^	0	0.00%	10	20%	10	20%		
Mortality	Alive	5	10%	5	10%	10	20%	0.023	0.887
	Died	19	38%	21	42%	40	80%		

* Significant relation.

**Table 2:** The relation of tumor glycolytic activity to the treatment modality as well as to patient and tumor characteristics and mortality.

		Tumor Glycolytic Activity Gradient Segmentation > 92 g		Gradient Segmentation ≤ 92 g		Total			
		N	%	N	%	N	N	*X* ^2^	*p*-Value
Age	<60	9	18%	12	24%	21	42%	0.381	0.536
	>60	15	30%	14	28%	29	58%		
Sex	Male	13	26%	15	30%	28	56%	0.063	0.802
	Female	11	22%	11	22%	22	44%		
ECOG performance status	0	2	4%	2	4%	4	8%	0.013	0.933
	1	22	44%	24	48%	35	92%		
Tumor Stage	II	0	0%	3	6%	3	6%	5.469	0.077 *
	III	10	20%	12	24%	22	44%		
	IV	14	28%	11	22%	25	50%		
Tumor Grade	Grade I	0	0%	0	0.00%	0	0%	5.256	0.033 *
	Grade II	7	14%	13	26%	20	40%		
	Grade III	17	34%	13	26%	30	60%		
Therapy	None	2	4%	1	2%	3	6%	5.786	0.022 *
	Chemotherapy	10	20%	5	10%	15	30%		
	Chemoradiotherapy	12	24%	17	34%	29	58%		
	Chemoradiotherapy + surgery	0	0	3	6	3	6%		
SUVmax	>6.5	17	34%	9	18%	26	52%	6.562	0.010 *
	≤6.5	7	14%	17	34%	24	48%		
50% SUVmax threshold	>82	18	36%	5	10%	23	46%	15.629	0.001 *
	≤82	6	12%	21	42%	27	54%		
metabolic tumor volume	<3 cm^3^	3	6%	7	14%	10	20%	5.409	0.020 *
	≥3 cm^3^–5 cm^3^	6	12%	10	20%	16	32%		
	>5 cm^3^–8 cm^3^	8	16%	6	12	14	28%		
	>8 cm^3^	7	14%	3	6%	10	20%		
Mortality	Alive	1	2%	9	18%	10	20%	7.231	0.007 *
	Died	23	46%	17	34%	40	80%		

* Significant relation.

**Table 3:** Survival time means and medians.

Group	Mean				Median			
	Estimate	Std. Error	95% CI		Estimate	Std. Error	95% CI	
			Lower Bound	Upper Bound			Lower Bound	Upper Bound
Peak SUV < 3.9	35.877	3.334	29.343	42.412	35.000	1.089	32.865	37.135
Peak SUV > 3.9	24.092	1.319	21.507	26.677	23.000	0.543	21.936	24.064
Overall	30.157	2.089	26.063	34.252	28.000	3.786	20.580	35.420

*p* value = 0.001.

**Table 4:** Survival time means and medians.

Group 2	Mean				Median			
	Estimate	Std. Error	95% CI		Estimate	Std. Error	95% CI	
			Lower Bound	Upper Bound			Lower Bound	Upper Bound
Gradient segmentation < 92 g	34.915	3.564	27.929	41.901	35.000	1.483	32.094	37.906
Gradient segmentation > 92 g	25.365	1.372	22.676	28.054	23.000	0.667	21.693	24.307
Overall	30.157	2.089	26.063	34.252	28.000	3.786	20.580	35.420

*p* value = 0.001.

**Table 5:** Survival time means and medians.

Group	Mean				Median			
	Estimate	Std. Error	95% CI		Estimate	Std. Error	95% CI	
			Lower Bound	Upper Bound			Lower Bound	Upper Bound
Peak SUV < 3.9	40.216	2.785	37.572	48.546	39.000	3.990	31.180	46.820
Peak SUV > 3.9	31.586	1.865	28.710	36.410	34.000	1.948	30.183	37.817
Overall	37.831	1.878	34.151	41.512	35.000	1.260	32.531	37.469

*p* value = 0.007.

**Table 6:** Survival time means and medians.

Group 2	Mean				Median			
	Estimate	Std. Error	95% CI		Estimate	Std. Error	95% CI	
			Lower Bound	Upper Bound			Lower Bound	Upper Bound
Gradient segmentation < 92 g	43.150	2.802	37.658	48.643	39.000	6.472	26.314	51.686
Gradient segmentation > 92 g	32.419	1.935	28.626	36.211	34.000	1.822	30.429	37.571
Overall	37.831	1.878	34.151	41.512	35.000	1.260	32.531	37.469

*p* value = 0.004.

**Table 7:** Correlation between serum MMP-2 and the patients’ characteristics.

		Serum MMP-2						Chi-Square	
		Normal Group (*n* = 38)		High Group (*n* = 12)		Total			
		N	%	N	%	N	%	*X* ^2^	*p*-Value
Age (years)	<60	15	39.5	6	50	21	42%	0.095	0.758
	>60	23	60.5	6	50	29	58%		
Sex	Male	18	47.4	5	41.7	23	46%	0.12	0.73
	Female	20	52.6	7	58.3	27	54%		
ECOG performance status	0	13	34.2	0	0	13	26%	5.548	0.019 *
	1	25	65.8	12	100	37	%74		
Tumor Stage	II	2	5.3	1	8.3	3	6%	6.267	0.043 *
	III	25	65.8	3	25	28	56%		
	IV	11	29	8	66.7	19	38%		
Tumor Grade	Grade I	7	18.4	3	25	10	20%	0.747	0.689
	Grade II	18	47.4	4	33.3	22	44%		
	Grade III	13	34.2	5	41.7	18	36%		
Therapy	None	2	5.3	1	8.3	3	6%	0.883	0.83
	chemotherapy	7	18.4	3	25	10	20%		
	chemoradiotherapy	18	47.4	6	50	14	28%		
	Chemoradiotherapy + surgery	11	28.9	2	16.7	13	26%		
glycolytic activity	gradient segmentation > 92 g	14	36.8	9	75	23	46%	5.346	0.021 *
	gradient segmentation ≤ 92 g	24	63.2	3	25	27	54%		
50% SUVmax threshold	>82	13	31.6	10	83.3	23	46%	8.860	0.003 *
	≤82	25	36.8	2	16.7	27	54%		
metabolic tumor volume	<3 cm^3^	9	23.7	1	8.3	10	20%	2.332	0.506
	≥3 cm^3^–5 cm^3^	17	44.7	7	58.3	24	48%		
	>5 cm^3^–8 cm^3^	9	23.7	2	16.7	11	22%		
	>8 cm^3^	3	7.9	2	16.7	5	10%		

* Significant relation.

## Data Availability

Data used and/or analyzed during this study are not available for public access because of patient privacy concerns but are available from the corresponding author upon reasonable request.
